# Adaptive reprogramming of carbon–nitrogen metabolism in *Klebsiella aerogenes* under nitrate-rich conditions

**DOI:** 10.3389/fmicb.2026.1859352

**Published:** 2026-06-17

**Authors:** Yanyan Chen, Meiqi Zhong, Minghan Lu, Jinpeng Huang, Hongrong Liang, Haonan Zou, Lele Xu, Yongqin Li

**Affiliations:** 1 School of Life Sciences and Technology, Lingnan Normal University, Zhanjiang, Guangdong, China; 2 Western Guangdong Provincial Engineering Technology Research Center of Seafood Resource Sustainable Utilization, Lingnan Normal University, Zhanjiang, Guangdong, China

**Keywords:** amino acid metabolism, aquatic microbiology, *Klebsiella aerogenes* B23, metabolomics, TCA cycle

## Abstract

**Introduction:**

*Klebsiella aerogenes* B23, a newly isolated marine strain, exhibits efficient nitrate assimilation and denitrification with minimal intermediate accumulation.

**Methods:**

This study examined its physiological and metabolomic responses to nitrogen‑deficient (DM1) and nitrate‑rich (DM2) conditions using nitrogen balance analysis and metabolomic profiling.

**Results:**

Nitrogen balance analysis showed that 83.28% of supplied nitrate in DM2 was incorporated into biomass, with 16.16% released as gaseous nitrogen. Metabolomic profiling revealed upregulation of TCA cycle intermediates (e.g., α‑ketoglutaric acid, succinic acid) and amino acids (e.g., lysine, arginine) under DM2, indicating enhanced carbon flux toward nitrogen assimilation. Downregulation of isocitric acid and acetyl‑CoA suggested rerouting of carbon skeletons to support active nitrogen utilization. KEGG pathway analysis highlighted lysine degradation, glyoxylate metabolism, and taurine pathways in facilitating this metabolic shift.

**Discussion:**

These findings demonstrate that *K. aerogenes* B23 efficiently couples energy production with nitrogen assimilation, offering potential for sustainable nitrate removal in aquaculture and wastewater treatment. Further proteomic studies may clarify the molecular basis of its versatile nitrogen metabolism.

## 1 Introduction

Microbial nitrogen metabolism is a cornerstone of global biogeochemical cycles, critically shaping both aquatic ecosystem productivity and industrial-scale wastewater treatment efficiency (Kuypers et al., 2018; Shaw et al., 2024; He et al., 2026). Building on this broader significance, it is notable that, within the family *Enterobacteriaceae*, members of the genus *Klebsiella* stand out for their adaptability and broad metabolic potential, particularly in nitrogen assimilation and denitrification pathways. For instance, *Klebsiella pneumoniae* has been reported to effectively remove nitrate and nitrite in contaminated waters, making it a target for biotechnological applications aimed at curbing eutrophication and mitigating pollution (Tomulescu et al., 2021). By comparison, similar capacities have been documented in *K. aerogenes*, a closely related species known to share many of the same fundamental metabolic traits (Chen et al., 2024). Recent research has highlighted *K. aerogenes* B23—a newly isolated marine strain—as a promising candidate for managing nitrogen-rich effluents, especially in aquaculture settings where elevated nitrogen levels can threaten water quality (Chen et al., 2023). Unlike many other denitrifying bacteria in this group, *K. aerogenes* B23 exhibits an ability to incorporate external nitrate directly into biomass without generating appreciable levels of intermediate byproducts, thus minimizing secondary pollution. Taken together, these distinctive features underscore its potential in environmentally sustainable approaches to nitrogen removal and water remediation.

Building on the recognition of B23’s unique advantages, it is also important to acknowledge that the family *Enterobacteriaceae* harbors various *Klebsiella* species with parallel adaptive traits for nitrogen utilization under marine or brackish conditions (Brisse et al., 2006). Recent studies have highlighted, for example, that *Klebsiella pneumoniae* strains isolated from coastal environments exhibit robust denitrification and nitrate assimilation pathways, contributing to improved water quality in aquaculture systems (Ren et al., 2024). In a similar vein, *K. aerogenes*—formerly classified as *Enterobacter aerogenes*—has emerged as another promising marine isolate, renowned for its adaptive metabolic traits and capacity to thrive across varying salinities (Brisse et al., 2006). Beyond its inherent denitrification capacity, *K. aerogenes* B23, a novel strain obtained from a marine aquaculture region, can strategically reroute carbon skeletons and reducing power (e.g., NADPH) toward amino acid and nucleotide biosynthesis under nitrate-rich conditions, showcasing a powerful model for microbial reprogramming (Chen et al., 2024). Such metabolic plasticity underscores the strain’s potential in addressing multiple nitrogen management challenges prevalent in aquaculture and industrial wastewater scenarios (Fan and Sun, 2024). Specifically, *K. aerogenes*’s preferential conversion of nitrate into biomass, accompanied by orchestrated changes in the TCA cycle and amino acid pathways, suggests a mechanism for boosting growth while minimizing the accumulation of intermediates like nitrite (Chen et al., 2024; Senior, 1975). Notably, comparable observations have also been reported in other *Klebsiella* sp. isolated from marine habitats, which exhibit high levels of denitrification efficiency and adaptability (Feng et al., 2018). Consequently, this capacity to optimize nitrogen flux has made *K. aerogenes* B23 an attractive candidate for advanced water treatment technologies, bioremediation efforts, and the sustainable expansion of aquaculture systems. By systematically leveraging its metabolic versatility, researchers and practitioners can further develop low-impact strategies to mitigate nitrogen pollution while maintaining productivity in marine-related industries.

To further dissect the pathways underlying these versatile capabilities, metabolomics has emerged as an essential tool for profiling intracellular and extracellular metabolites under different environmental conditions—such as variations in salinity, temperature, and nutrient availability (Liebeke and Lalk, 2014; Huang et al., 2025). Recent marine metabolomics studies have underscored the integral link between carbon metabolism and nitrogen utilization in various microbial taxa (Smith et al., 2019). For example, targeted and untargeted metabolomic analyses of *Prochlorococcus* and *Synechococcus*—two dominant marine cyanobacterial genera—revealed that when exposed to nitrogen-limited or nitrogen-enriched conditions, these organisms undergo pronounced alterations not only in amino acid and nucleotide biosynthetic pathways but also in the abundance of key TCA cycle intermediates (Díez et al., 2023; Moreno-Cabezuelo et al., 2023). Accumulation or depletion of metabolites such as *α*-ketoglutarate, succinate, and malate can have a cascading impact on NAD(P)H regeneration and glutamate/glutamine synthesis, thereby modulating the cells’ overall nitrogen assimilation capacity (Yuan et al., 2009). While these insights from cyanobacteria illuminate nitrogen responses in photosynthetic microbes, investigations into heterotrophic marine bacteria—including several *γ*-proteobacteria isolates—highlight the importance of cofactor biosynthesis, polyamine production, and TCA flux in driving efficient nitrogen scavenging (Delmont et al., 2022; Zheng et al., 2020). For instance, upregulation of polyamine biosynthesis pathways (e.g., spermidine, putrescine) has been linked to enhanced cellular resilience and growth rates under nitrogen-stress conditions, while coordinated shifts in TCA intermediates frequently coincide with augmented ammonium or nitrate uptake (Romero et al., 2018). Taken together, these integrated responses suggest that energy metabolism and amino acid pathways operate synergistically to maintain cellular homeostasis, allocating carbon skeletons and reducing power according to the availability or limitation of nitrogen (Dang and Chen, 2017; Dawson et al., 2023; Chen et al., 2026). Collectively, these studies emphasize that metabolite profiling offers a crucial window into the regulatory checkpoints that govern marine microbial adaptability, illuminating specific metabolic nodes—particularly within energy and amino acid biosynthesis routes—that might remain cryptic if only transcriptomic or proteomic data were employed (Goulitquer et al., 2012).

Building upon these metabolomic insights, the present study aims to elucidate the intricate biochemical and regulatory mechanisms by which *K. aerogenes* B23 orchestrates its metabolism under nitrogen-deficient versus nitrate-rich settings. By combining a metabolomic overview with nitrogen balance assessments, we provide a comprehensive understanding of how this bacterium effectively couples energy production to nitrogen assimilation, thereby highlighting its potential for high-efficiency nitrate removal. Ultimately, the insights gained here could pave the way for advanced strategies in aquaculture water management and the broader field of bioremediation, especially given the pressing need to mitigate excess nitrogen in aquatic systems.

## 2 Materials and methods

### 2.1 Bacterium and media

*Klebsiella aerogenes* B23, isolated from Zhanjiang Yuehai Aquatic Seeding Company Limited on Donghai Island in Zhanjiang, China (Chen et al., 2023), was used in this study. The denitrification media (DM1 and DM2) were prepared with the following components per liter of distilled water: 5.0 g of sucrose, 1.0 g of K_2_HPO_4_, 1.0 g of KH_2_PO_4_, 5.0 g of NaCl, and 2 mL of a trace element solution, which contained 1.1 g of MnSO_4_, 1.0 g of MgSO_4_, 1.6 g of CuSO_4_, and 1.8 g of FeSO_4_ per liter of distilled water. DM1 excluded KNO_3_, whereas DM2 included 0.36 g of KNO_3_ as the sole nitrogen source. For comparison, the Luria–Bertani (LB) medium was composed of 10 g of peptone, 5 g of yeast extract, and 5 g of NaCl per liter of distilled water. Additionally, 1 × phosphate-buffered saline (PBS) solution was obtained from BioSharp Biotech (Beijing, China). All media were sterilized via autoclaving at 121 °C for 20 min before use.

### 2.2 Experimental design and sample collection

*Klebsiella aerogenes* B23 was first activated and cultured in LB broth until it reached the logarithmic growth phase (OD_600_ = 1.0). LB medium was used only for initial pre-culture to obtain a consistent seed culture; all experimental analyses (growth, nitrogen balance and metabolomics) were performed after transferring cells to DM1 or DM2. Afterward, the bacterial cells were washed three times with PBS and resuspended. This suspension was then inoculated into DM1 or DM2 at a 5% (v/v) inoculum ratio and sealed with breathable sealing films. The inoculated flasks were incubated in a shaking incubator at 30 °C for 48 h. At the end of the incubation period, bacterial samples were collected by centrifugation at 24,200 × g for 10 min and then stored at −80 °C. These samples were subsequently sent to Metware Biotechnology Co., Ltd. (Wuhan, China) for metabolomic analysis.

Following inoculation into DM1 and DM2, the bacterial growth (OD_600_) was monitored using a UV-3600PLUS ultraviolet spectrophotometer (SHIMADZU, China). In addition, nitrogen utilization (including total nitrogen, nitrate, ammonium and nitrite) was measured at intervals of 0, 2, 6, 12, 24 and 48 h. The concentrations of total nitrogen, nitrate, ammonium and nitrite were determined using alkaline potassium persulfate photometry, phenol disulfonic acid photometry, Nessler’s reagent spectrophotometry and N-(1-naphthyl)ethylenediamine photometry, respectively, following the methods of Gilcreas (1966). All experiments were performed in triplicate to ensure reproducibility.

### 2.3 UPLC and ESI-MS/MS conditions

Sample extracts were analyzed using a Waters ACQUITY H-Class UPLC system coupled with a QTRAP® 6,500 + MS (Sciex). Separation was performed on an ACQUITY UPLC BEH Amide column (2.1 × 100 mm, 1.7 μm) using an amide-based method. The solvent system consisted of 10 mM ammonium acetate with 0.3% ammonium hydroxide (A) and 90% acetonitrile/water (v/v) (B). The gradient elution was as follows: 95% B for 0–1.2 min, decreased to 70% B by 8 min, to 50% B by 11 min, and reverted to 95% B by 15 min, at a flow rate of 0.4 mL/min and a column temperature of 40 °C. An injection volume of 2 μL was used. The QTRAP® 6,500 + system operated with an ESI Turbo Ion Spray interface, acquiring LIT and QQQ scans in both positive and negative ion modes via Analyst 1.6.3 software. ESI source parameters included a temperature of 550 °C, ion spray voltages of +5,500 V and −4,500 V, and a curtain gas pressure of 35 psi. Metabolites involved in energy metabolism were evaluated using scheduled multiple reaction monitoring (MRM). Quantification was performed with Sciex Multiquant 3.0.3, with MRM transitions optimized based on metabolite elution profiles.

### 2.4 Data pre-processing and quality control (QC)

Initial data processing was conducted using MultiQuant 3.0.3, followed by peak alignment, retention time correction, and peak area extraction with XCMS software. Quality control was ensured through total ion chromatogram (TIC) comparisons, principal component analysis (PCA), sample correlation analysis, and evaluation of zero-centered data (Ctr) and coefficient of variation (CV).

### 2.5 Differential metabolite screening and bioinformatics analysis

Differential metabolites between groups were identified using univariate methods (fold change [FC] analysis and t-test) and multivariate techniques (PCA). Metabolites were considered significant if VIP > 1, *p* < 0.05, and FC > 2 or ≤ 0.5. Correlation and KEGG pathway analyses were performed to assess the relationships and functions of these metabolites. Cluster analysis, with unit variance scaling, was used to visualize changes in metabolite levels.

### 2.6 Statistical analysis

All experiments were performed with three independent biological replicates (𝑛=3). Results are presented as mean ± standard deviation (SD). Statistical analyses were performed using SPSS 19.0 (SPSS Inc., Chicago, IL, USA). Differences between treatments were evaluated using t-tests, with significance set at *p* < 0.05.

## 3 Results and analysis

### 3.1 Nitrogen balance analysis

**Figure 1:**
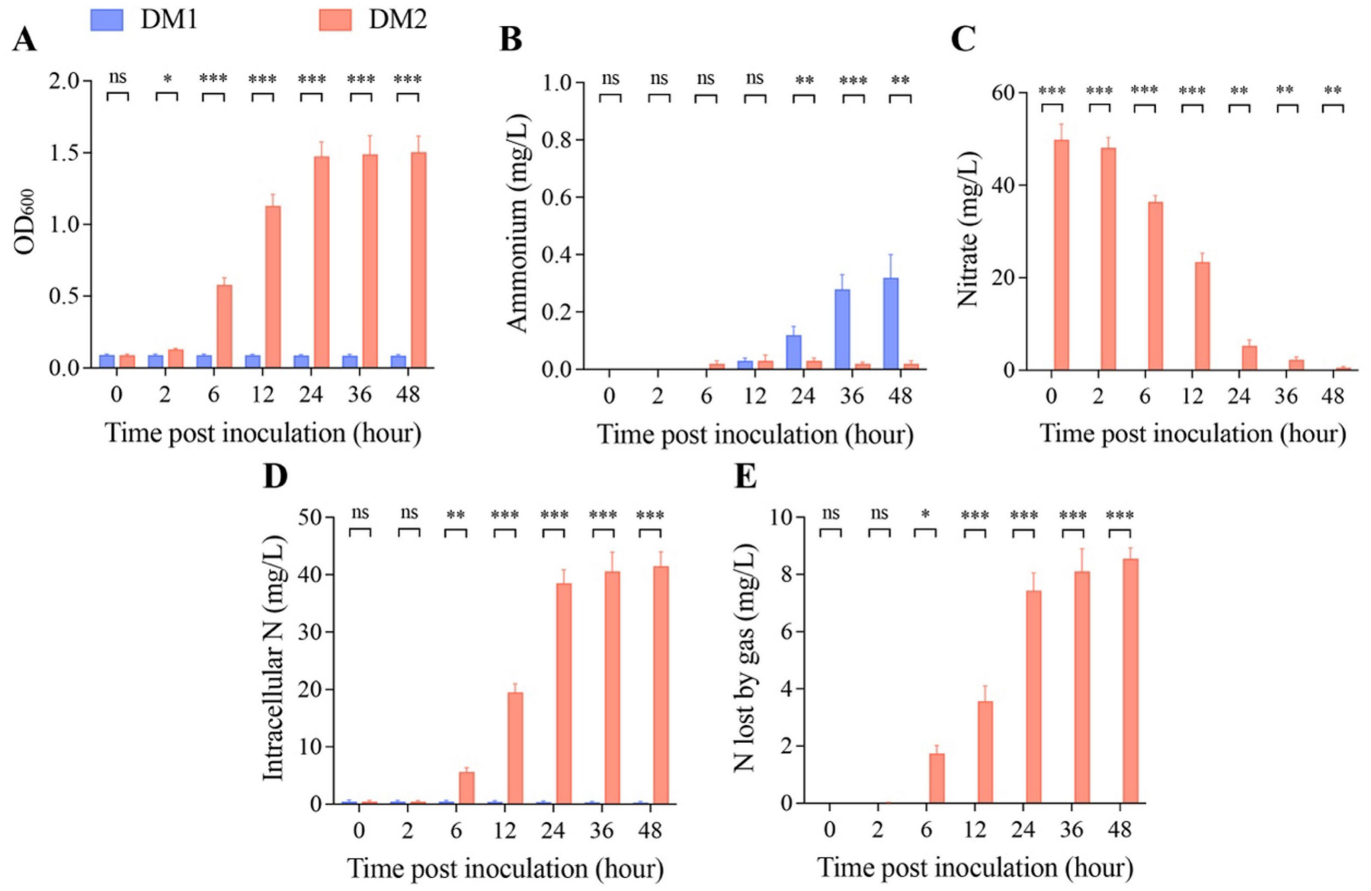
The growth and nitrogen balance of *K. aerogenes* B23 during the denitrification process (units: mg/L). **(A)** OD_600_ value; **(B)** ammonium concentration; **(C)** nitrate concentration; **(D)** intracellular nitrogen concentration; **(E)** nitrogen lost as gas, calculated as total nitrogen (0 h) – total nitrogen (48 h). The label “*” indicates *p* < 0.05, “**” indicates *p* < 0.01, and “***” indicates *p* < 0.001.

In this study, we evaluated nitrogen balance by monitoring and calculating changes in different nitrogen forms (Figure 1). The findings showed that *K. aerogenes* B23 experienced significantly restricted growth in DM1, likely due to the lack of an external nitrogen source. After 48 h, only trace amounts of ammonium and a minor decrease in intracellular nitrogen were observed, possibly resulting from residual nitrogen in the cytoplasm of some cells. In contrast, *K. aerogenes* B23 exhibited rapid growth under DM2 conditions, accompanied by substantial nitrate consumption. The trends in intracellular nitrogen accumulation and gaseous nitrogen loss closely mirrored the bacterial growth pattern. Nitrite levels were undetectable in both DM1 and DM2 throughout the experiment. Notably, 83.28% of the supplied nitrate was assimilated into intracellular nitrogen, while 16.16% was converted into gaseous nitrogen and released.

### 3.2 QC of LC–MS data

We performed an overlapping display analysis of total ion current (TIC) plots for identical QC samples. The substantial overlap in TIC curves indicated consistent retention times and stable peak intensities, confirming both the reliability of the mass spectrometry data and the robust signal stability of our platform. The consistency of these results further suggested minimal instrument-related error when identical samples were analyzed across treatments (Supplementary Figures S1, S2).

Pearson correlation analysis of the QC samples revealed satisfactory stability throughout the entire detection period, underscoring high data quality (Supplementary Figures S3A, S4A). Meanwhile, the coefficient of variation (CV) highlighted the data’s dispersion, with 80% of the QC samples presenting a CV below 0.2, reinforcing the experiment’s integrity and the reliability of the findings (Supplementary Figures S3B, S4B). PCA also illustrated clear distinctions in metabolome profiles across groups, reflecting significant differences under various treatments (Supplementary Figures S3C, S4C).

### 3.3 Quantification and classification of identified metabolites

**Figure 2:**
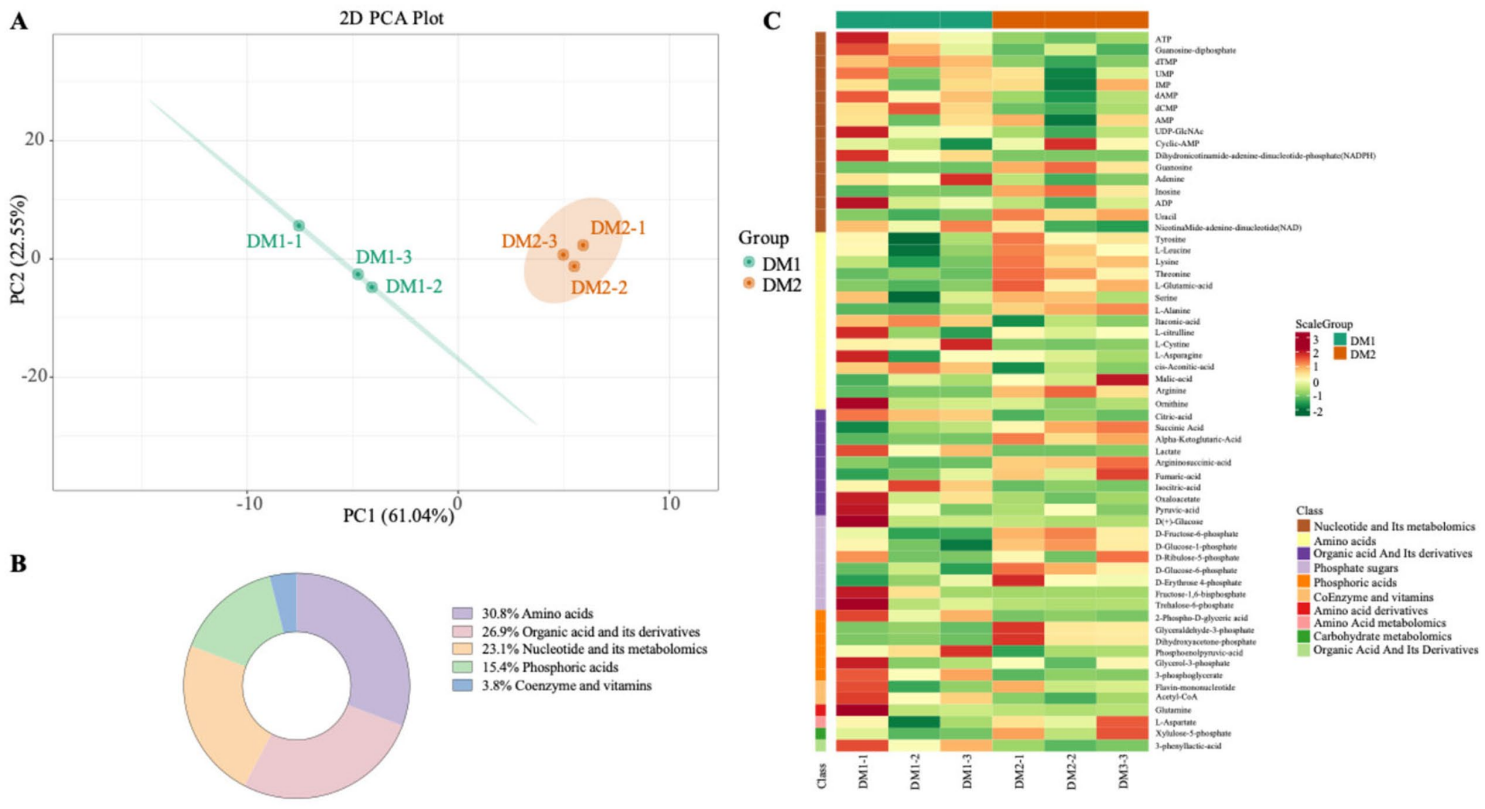
Comparative classification of energy-related metabolite of *K. aerogenes* B23 under KNO_3_ treatment. **(A)** Quality control of metabolomics data; **(B)** Classification of metabolites under KNO_3_ stress; **(C)** Heatmap showed the results of the clustering analysis of differential metabolites.

**Figure 3:**
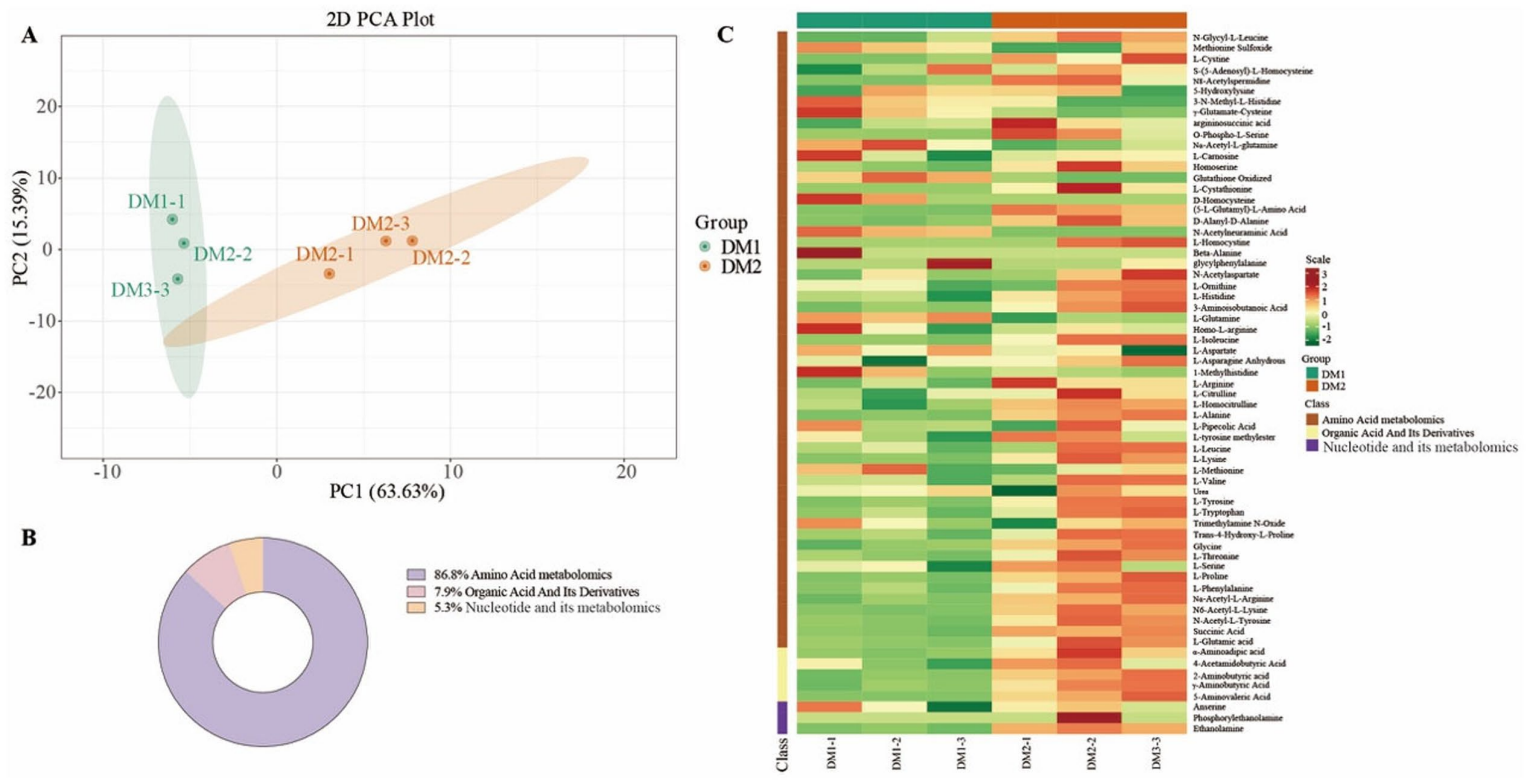
Comparative classification of amino acid-related metabolite of *K. aerogenes* B23 under KNO_3_ treatment. **(A)** Quality control of metabolomics data; **(B)** Classification of metabolites under KNO_3_ stress; **(C)** Heatmap showed the results of the clustering analysis of differential metabolites.

We examined the dynamic metabolite profiles and observed a clear separation between DM1 and DM2 samples via PCA (Figures 2A, 3A). Orthogonal partial least squares discriminant analysis (OPLS-DA) further validated these differences, as indicated by distinct clustering in the corresponding score plots (Supplementary Figures S5). Within the energy-related group, 5 metabolites were identified in positive ion mode and 21 in negative ion mode, with 30.8% classified as amino acids, 26.9% as organic acids or derivatives, and 23.1% as nucleotides or related compounds (Figure 2B). Within the amino acid–related group, 36 were detected in positive ion mode and 2 in negative ion mode, with 86.8% classified as amino acids and 7.9% as organic acids or derivatives (Figure 3B). Most metabolites showed higher abundances under DM2 treatment, a pattern further highlighted by unit variance scaling (Figures 2C, 3C).

### 3.4 Differential metabolite categorization and correlation

**Figure 4:**
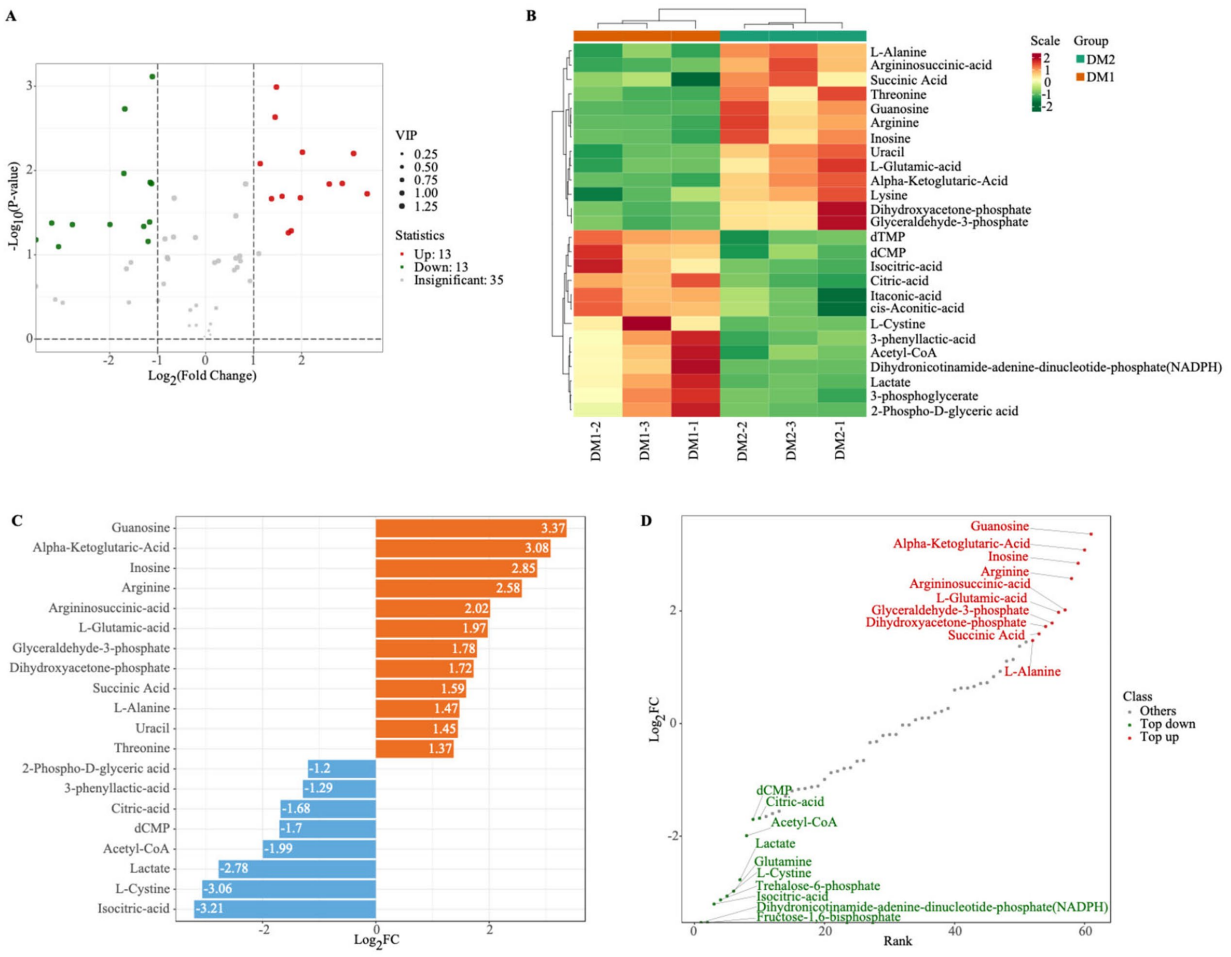
Up- and down-regulation of energy-related differential metabolites in *K. aerogenes* B23 under KNO_3_ treatment. **(A)** Volcano plots showing the up-regulate and down-regulated energy-related metabolites; **(B)** Heatmap showing the up- and down-regulated energy-related metabolites; **(C)** The bar chart of energy-related differential multiples; **(D)** Dynamic distribution of energy-related differential metabolic content.

**Figure 5:**
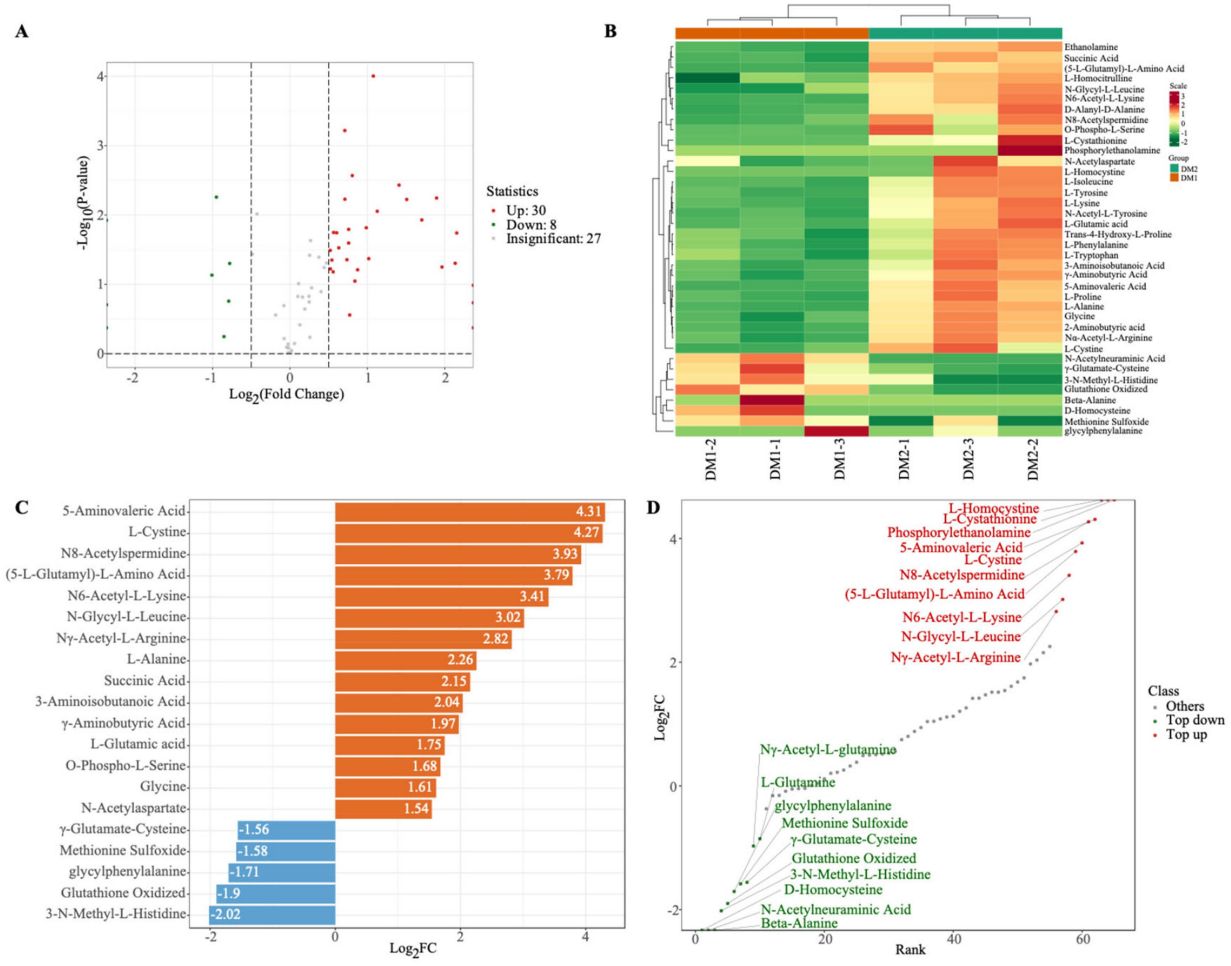
Up- and down-regulation of amino acid-related differential metabolites in *K. aerogenes* B23 under KNO_3_ treatment. **(A)** Volcano plots showing the up-regulate and down-regulated amino acid-related metabolites; **(B)** Heatmap showing the up- and down-regulated amino acid-related metabolites; **(C)** The bar chart of amino acid-related differential multiples; **(D)** Dynamic distribution of amino acid-related differential metabolic content.

Volcano plot analysis identified 26 differential energy-related metabolites between DM1 and DM2, with 13 upregulated and 13 downregulated (Figure 4A). Upregulated metabolites—including lysine, threonine, L-glutamic acid, L-alanine, arginine, guanosine, inosine, uracil, succinic acid, alpha-ketoglutaric acid, argininosuccinic acid, glyceraldehyde-3-phosphate, and dihydroxyacetone phosphate—may be particularly relevant under nitrate-supplemented conditions (Supplementary Table S1). In total, 38 significant differential amino acid–related metabolites were identified between DM1 and DM2, with 30 upregulated and 8 downregulated (Figure 5A). The upregulated metabolites included 5-aminovaleric acid, L-cystine, N8-acetylspermidine, (5-L-glutamyl)-L-amino acid, N6-acetyl-L-lysine, N-glycyl-L-leucine, Nα-acetyl-L-arginine, L-alanine, succinic acid and 3-aminoisobutanoic acid (Supplementary Table S2). These may perform positive functions under sole nitrogen conditions.

A heatmap of these differential metabolites indicated pronounced differences among the comparison groups (Figures 4B, 5B). Based on qualitative and quantitative analyses of the detected energy-related metabolites, the top five upregulated metabolites—guanosine, alpha-ketoglutaric acid, inosine, arginine and argininosuccinic acid—and the top five downregulated metabolites—lactate, isocitric acid, L-cystine, acetyl-CoA and dCMP—displayed the most notable changes (Figure 4C). The top five upregulated amino acid–related metabolites were 5-aminovaleric acid, L-cystine, N8-acetylspermidine, (5-L-glutamyl)-L-amino acid and N6-acetyl-L-lysine, while the top five downregulated amino acid–related metabolites were 3-N-methyl-L-histidine, oxidized glutathione, glycylphenylalanine, methionine sulfoxide and *γ*-glutamate-cysteine (Figure 5C).

Based on the dynamic distribution plot of metabolite content differences, the top five upregulated energy-related metabolites in the DM2 vs. DM1 comparison were guanosine, alpha-ketoglutaric acid, inosine, arginine and argininosuccinic acid, whereas the most highly downregulated included fructose-1,6-bisphosphate, NADPH, isocitric acid, trehalose-6-phosphate, L-cystine and glutamine (Figure 4D). The top five upregulated amino acid–related metabolites were L-homocystine, L-cystathionine, phosphorylethanolamine, 5-aminovaleric acid and L-cystine, whereas the top five downregulated amino acid–related metabolites were beta-alanine, N-acetylneuraminic acid, D-homocysteine, 3-N-methyl-L-histidine and oxidized glutathione (Figure 5D).

### 3.5 K-means cluster analysis of differential metabolites

**Figure 6:**
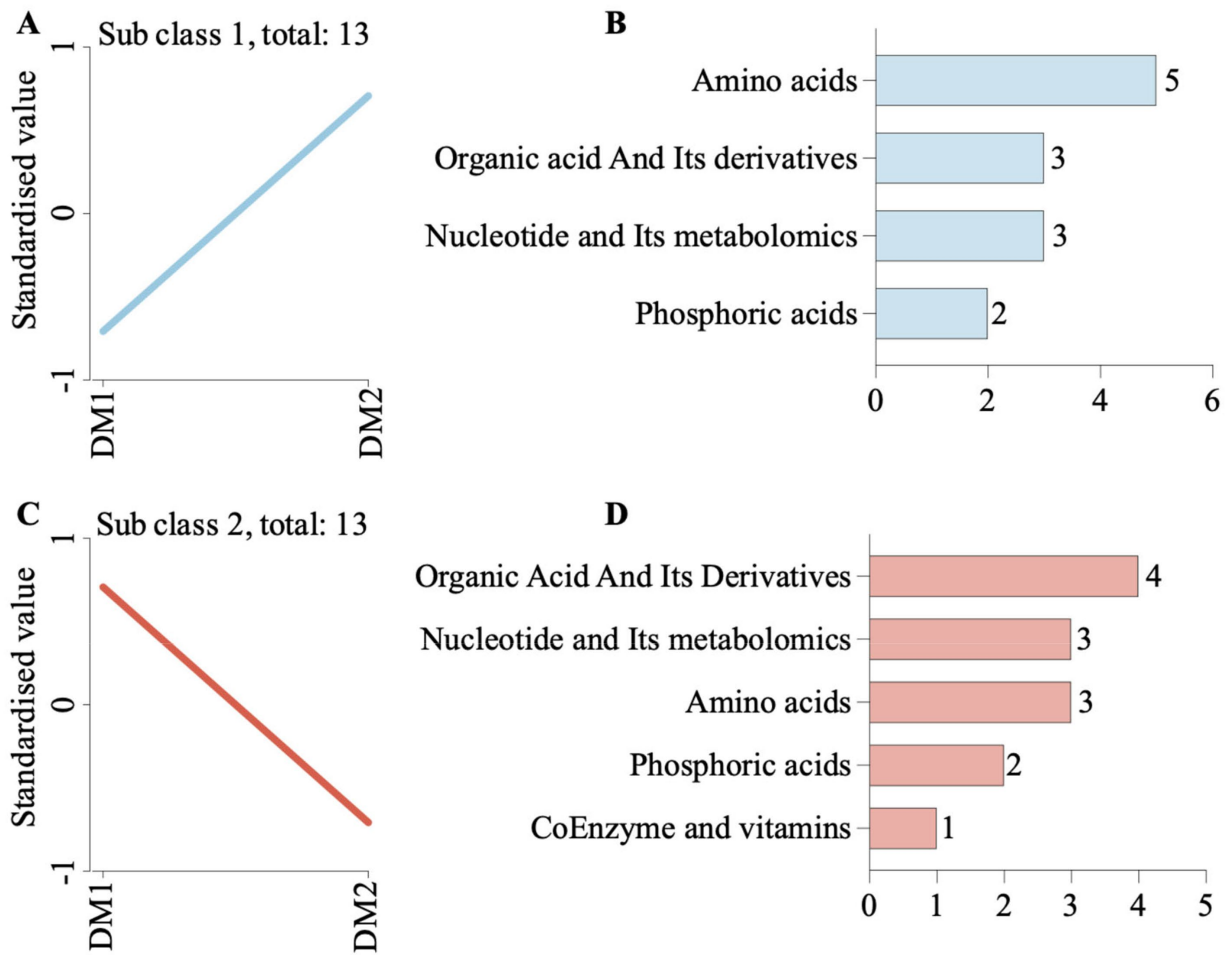
K-means cluster analysis of energy-related differential metabolites and their metabolite categories. **(A)** Expression trends of Class 1 metabolites; **(B)** Expression trends of Class 2 metabolites; **(C)** Distribution of compound classes in Class 1; **(D)** Distribution of compound classes in Class 2.

To investigate metabolite variation under KNO_3_ treatment, we performed K-means clustering on the 26 identified energy-related metabolites (Figure 6; Text 1). The resulting patterns formed two distinct classes. In Class 1, metabolite expression was consistently higher in DM1 than in DM2, encompassing 5 amino acids, 3 organic acids and derivatives, 3 nucleotides and related metabolites and 2 phosphoric acids. In contrast, Class 2 showed a different distribution, comprising 4 organic acids and derivatives, 3 nucleotides and related metabolites, 3 amino acids, 2 phosphoric acids and 1 coenzyme/vitamin. These findings underscore dynamic shifts in metabolite expression under KNO_3_ treatment and highlight their links to various compound classes.

### 3.6 KEGG and Pearson correlation analysis

**Figure 7:**
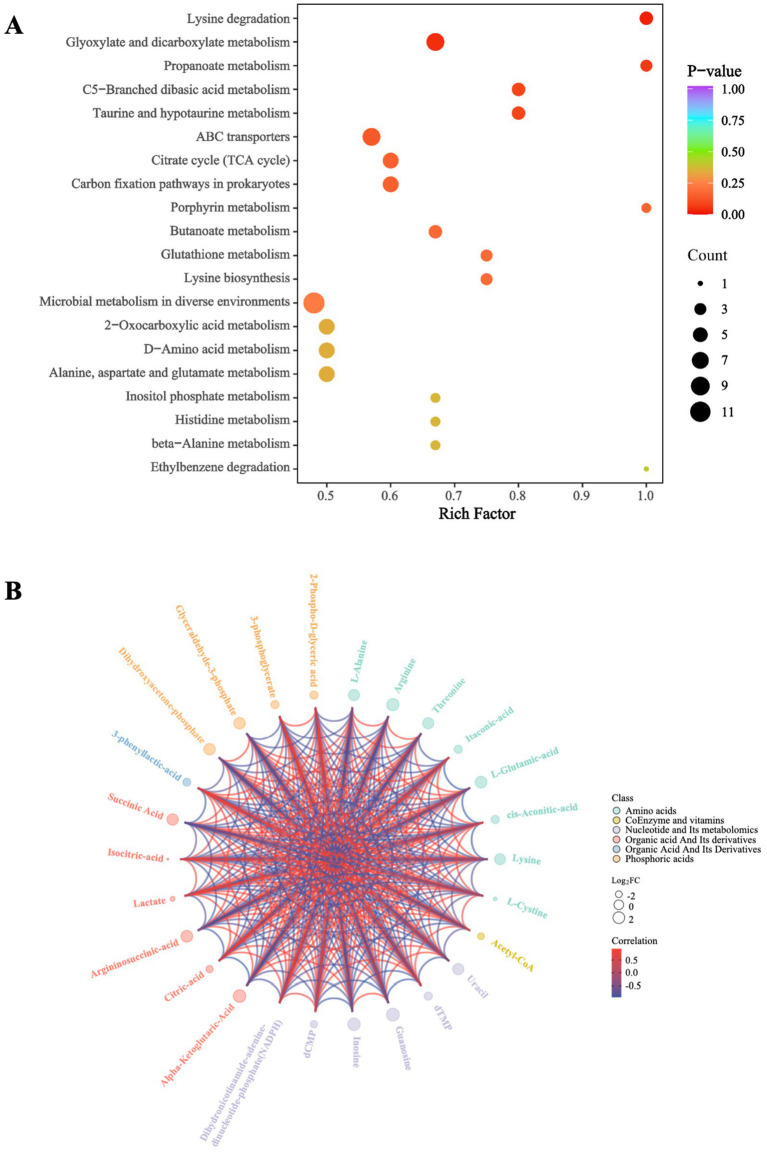
Energy-related differential metabolite enrichment pathways and their correlation analysis. **(A)** Differential metabolites were enriched KEGG pathway in DM2 vs. DM1 group; **(B)** the correlation analyzed the differential metabolites in DM2 vs. DM1 group.

**Figure 8:**
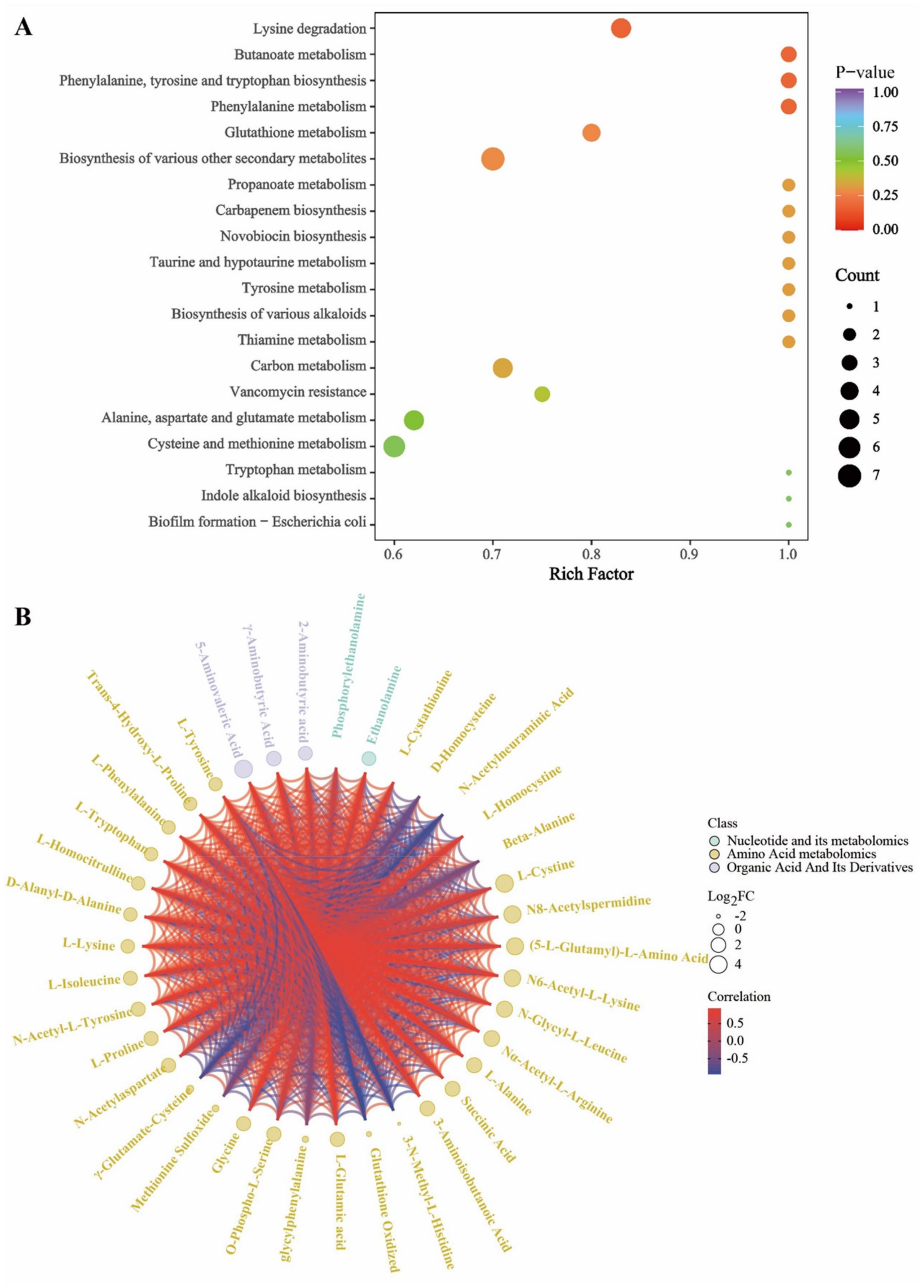
Amino acid-related differential metabolite enrichment pathways and their correlation analysis. **(A)** Differential metabolites were enriched KEGG pathway in DM2 vs. DM1 group; **(B)** the correlation analyzed the differential metabolites in DM2 vs. DM1 group.

Based on KEGG pathway analysis, the DM1 vs. DM2 comparison revealed significant enrichment of lysine degradation; glyoxylate and dicarboxylate metabolism; propanoate metabolism; C5-branched dibasic acid metabolism; and taurine and hypotaurine metabolism (Figure 7A). In addition, lysine degradation, butanoate metabolism, phenylalanine/tyrosine/tryptophan biosynthesis, phenylalanine metabolism, and glutathione metabolism were also significantly enriched pathways (Figure 8A).

Pearson correlation analysis indicated that metabolites in the same category showed positive correlations and similar accumulation patterns, whereas metabolites with opposing trends belonged to different classes (Figure 7B). In particular, arginine positively correlated with threonine, guanosine, and inosine in the DM1 vs. DM2 group, while alpha-ketoglutaric acid and argininosuccinic acid displayed positive correlations with threonine and L-alanine, respectively. L-cystathionine positively correlated with multiple metabolites, including N-glycyl-L-leucine, ethanolamine, D-alanyl-D-alanine, L-isoleucine, L-homocitrulline, L-lysine, trans-4-hydroxy-L-proline, N6-acetyl-L-lysine, N-acetyl-L-tyrosine, and L-glutamic acid in the DM1 vs. DM2 group (Figure 8B).

## 4 Discussion

Under nitrogen-deficient conditions (DM1), *Klebsiella aerogenes* B23 exhibited markedly limited growth, with only trace levels of ammonia and minimal intracellular nitrogen loss detected. This likely reflects the utilization of residual intracellular nitrogen to sustain basal metabolic activities (Chen et al., 2024). By contrast, in DM2, abundant nitrate supply not only accelerated bacterial proliferation but also substantially enhanced nitrogen assimilation and gaseous nitrogen production. The synchronized increases in intracellular nitrogen content and gas-phase nitrogen closely aligned with the growth curve, indicating that this strain efficiently exploits nitrate for both assimilation and denitrification (Huang et al., 2020; Li et al., 2021). Notably, 83.28% of the nitrate was incorporated into the cells, whereas only 16.16% was converted to gaseous nitrogen, with no nitrite accumulation observed throughout the process—a finding consistent with previous research on nitrate-reducing bacteria that rapidly convert nitrate without accumulating intermediates; moreover, studies on *Klebsiella* TCA-nitrogen coupling have shown that carbon flux reallocation via citrate synthase regulation can enhance nitrogen assimilation efficiency (Chen et al., 2023; Yoshidome et al., 2025). Overall, these results underscore the pivotal role of exogenous nitrate in driving *K. aerogenes* B23 growth and nitrogen turnover, offering promising avenues for its application in aquaculture and wastewater treatment.

An overlapping display of TIC chromatograms for identical QC samples revealed highly consistent retention times and peak intensities, confirming both the stability of instrument detection and the negligible measurement error, in accordance with established high-quality LC–MS protocols (Gika et al., 2012; Broadhurst et al., 2024). Likewise, PCA of the QC samples demonstrated excellent stability, with more than 80% of the QC replicates exhibiting CV below 0.2 (Supplementary Figures S3B, S4B), a criterion commonly used to assess data reproducibility in untargeted metabolomics studies (Gika et al., 2012), thereby reinforcing the reliability and reproducibility of these data. Furthermore, both PCA and OPLS-DA indicated significant metabolic distinctions between the DM1 and DM2 treatments, underscoring the critical role of exogenous nitrate in shaping the metabolite spectrum, a finding consistent with other multi-omics studies on nitrogen-mediated metabolic regulation (Bai et al., 2025; Mulaudzi et al., 2025). Consistent with previous findings that nitrogen availability can profoundly influence microbial energy metabolism and amino acid synthesis (Inabe et al., 2021; Huang et al., 2025), most energy- and amino acid–related metabolites were significantly elevated under the DM2 condition. Collectively, these rigorous quality-control measures and multivariate statistical analyses lay a robust foundation for pinpointing pivotal metabolic shifts, highlighting the indispensable utility of precise metabolomic approaches in elucidating microbial responses to varying nitrogen sources.

In this study, *K. aerogenes* B23 exhibited distinct metabolic responses under nitrogen-deficient (DM1) and nitrate-rich (DM2) conditions, as evidenced by significant shifts in both energy- and amino acid–related metabolites. Our data showed that TCA cycle intermediates—such as *α*-ketoglutaric acid (AKG), succinic acid and argininosuccinic acid—were upregulated under DM2, indicating enhanced carbon oxidation and energy generation to support increased growth and nitrogen assimilation (Foyer et al., 2011; He et al., 2026). In particular, the elevation of AKG underscores its dual role as both a key TCA intermediate and a crucial node in the glutamate–glutamine cycle, facilitating active nitrogen assimilation (Wu et al., 2016; Yan et al., 2026). This enhanced energetic output appears partly driven by the rerouting of carbon skeletons toward the late stages of the TCA cycle, as evidenced by the downregulation of isocitric acid and acetyl-CoA—two metabolites typically associated with upstream carbon flux (Corbet et al., 2016; Huang et al., 2022).

Parallel to these observations, nucleoside and nucleotide derivatives (e.g., guanosine, inosine) were also found at higher levels in DM2. This finding suggests that, once nitrogen is plentiful, the bacterium allocates additional resources to DNA/RNA synthesis for cell proliferation (Trautwein et al., 2018). Notably, NADPH was downregulated in DM2, potentially due to its heightened consumption in driving reductive biosynthesis of amino acids and nucleotides under nitrate-rich conditions (Schiffer et al., 2020). This is consistent with findings in *Clostridium thermocellum*, where a need to reoxidize NADPH was shown to drive amino acid secretion, and deletion of NADPH-supplying pathways significantly reduced branched-chain amino acid production (Yayo et al., 2023). The observed decrease in fructose-1,6-bisphosphate and other early glycolytic intermediates further supports the notion that glycolysis may accelerate under DM2, channeling more carbon toward TCA cycle intermediates and thereby expediting nitrogen incorporation into biomass (Foyer et al., 2011).

Amino acids and their derivatives displayed similarly pronounced shifts. Lysine, threonine, arginine and glutamic acid were all upregulated under DM2, corroborating previous findings that nitrogen-rich environments trigger intensification of amino acid biosynthesis (Sardans et al., 2020). Argininosuccinic acid, in particular, indicated activation of the urea cycle and enhanced interconversion between arginine and ornithine to manage excess nitrogen (Nagamani et al., 2012). Additionally, the accumulation of polyamine-related metabolites—such as 5-aminovaleric acid and N8-acetylspermidine—suggests a strategy to stabilize nucleic acids and cell membranes while maintaining intracellular osmotic balance (Miller-Fleming et al., 2015). This aligns with recent findings that increased arginine and ornithine availability actively promotes polyamine metabolism, substantially increasing key polyamines like putrescine, spermidine, and spermine via upregulation of ADC, ODC, SPDS and SPMS enzymes (Hamed et al., 2025). In contrast, certain metabolites (e.g., *β*-alanine, dCMP, and N-acetylneuraminic acid) decreased in DM2, implying that once surplus nitrate becomes available, the cell channels resources primarily into crucial amino acids and TCA-linked intermediates, downregulating secondary or stress-related pathways.

These metabolite-level alterations were further underscored by K-means clustering and KEGG pathway analyses, which identified significant enrichment in lysine degradation, glyoxylate and dicarboxylate metabolism, and propanoate metabolism. The strong correlations observed among amino acids, TCA intermediates, and nucleotide precursors highlight the dynamic interplay between carbon and nitrogen metabolism under nitrate-supplemented conditions (Zhou et al., 2024). Collectively, these findings align with prior reports on denitrifying and nitrate-assimilating bacteria, which show coordinated upregulation of TCA cycle intermediates, amino acid biosynthesis, and nucleic acid formation in response to exogenous nitrogen sources (Guo et al., 2024). Taken together, our results reveal a metabolically orchestrated adaptation of *K. aerogenes* B23 to nitrate availability. By reinforcing TCA cycle throughput, augmenting amino acid biosynthesis, and reallocating carbon and reducing equivalents (e.g., NADPH) toward nitrogen assimilation, the bacterium efficiently couples energy production with robust biomass formation. These insights not only deepen our understanding of microbial nitrogen metabolism but also suggest that *K. aerogenes* B23 could serve as a promising candidate for applications requiring effective nitrate removal or denitrification, such as aquaculture water treatment or wastewater bioremediation. Future work may focus on integrating transcriptomic and proteomic data to explore regulatory nodes and confirm the contribution of specific pathways, thereby advancing our knowledge of the precise molecular mechanisms underlying this versatile bacterium’s adaptability in nitrogen-rich environments.

Several potential caveats of this study should be acknowledged. First, our metabolomic data reflect steady-state metabolite levels, not metabolic flux; confirmation of carbon–nitrogen flux dynamics would require ^13^C/^15^N tracer experiments. Second, the comparison between DM1 (nitrogen-deficient) and DM2 (nitrate-rich) reflects the presence versus absence of an exogenous nitrogen source, rather than a direct comparison between different nitrogen forms (e.g., nitrate vs. ammonium). Third, the results were obtained under laboratory batch culture conditions; further studies in real aquaculture or wastewater systems are necessary to validate the strain’s practical applicability. Despite these limitations, our study provides a robust metabolomic framework for understanding carbon–nitrogen metabolic reprogramming in *K. aerogenes* B23 in response to nitrate-rich versus nitrogen-deficient conditions.

## 5 Conclusion

**Figure 9:**
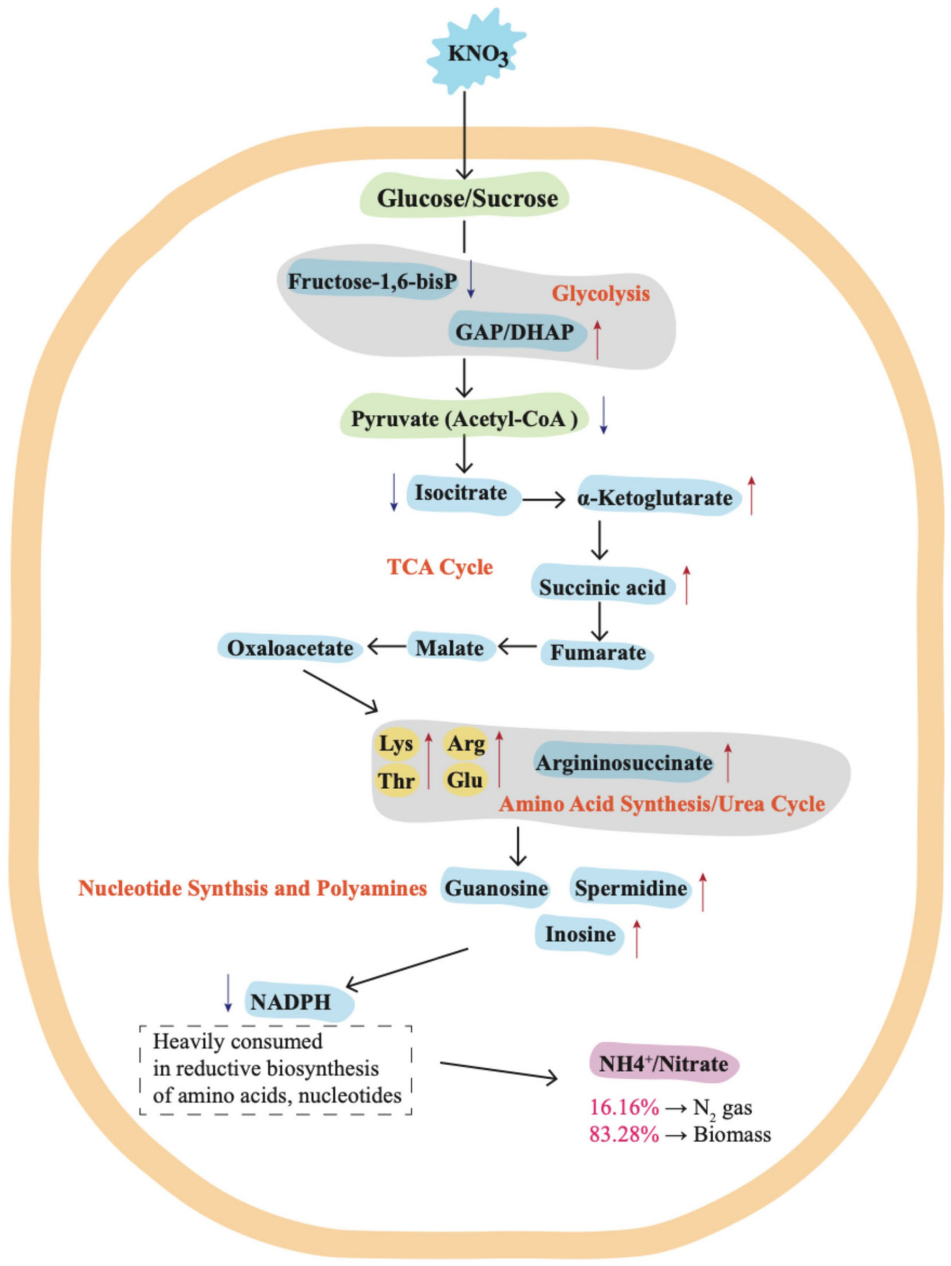
Patterns of KNO_3_ treatment affecting energy and amino acid metabolism in *K. aerogenes* B23.

In conclusion, under nitrate-rich conditions (DM2), *K. aerogenes* B23 markedly enhances TCA cycle flux, amino acid and nucleotide synthesis, and reallocates key carbon sources and reductants (e.g., NADPH) to achieve both efficient energy provision and robust biomass accumulation (Figure 9). Compared with the nitrogen-limited environment (DM1), DM2 cultivation significantly promotes bacterial growth, nitrogen assimilation, and denitrification, as evidenced by coordinated upregulation of energy metabolism and amino acid pathways at the metabolome level. These findings not only advance our understanding of microbial carbon–nitrogen coupling mechanisms but also underscore *K. aerogenes* B23’s potential for high-efficiency nitrate removal and water quality remediation. Future work integrating proteomic analyses will provide deeper insights into its core metabolic pathways and regulatory nodes, ultimately elucidating the molecular mechanisms underlying its adaptive and optimized metabolism in nitrogen-rich settings.

## Data Availability

The datasets presented in this study can be found in online repositories. The names of the repository/repositories and accession number(s) can be found at: doi: 10.6084/m9.figshare.28650260.

## References

[ref1] Bai X. Huang D. Yang W. Yue Y. Zhao N. Wang N. . (2025). Ammonium-induced microbial and metabolic activity in aerobic methane oxidation processes by zeolite-leaching systems. J. Environ. Chem. Eng. 13:118637. doi: 10.1016/j.jece.2025.118637

[ref2] Brisse S. Grimont F. Grimont P. A. D. (2006). “The Genus *Klebsiella*,” in The Prokaryotes, Eds. Dworkin M. Falkow S. Rosenberg E. Schleifer K.-H. Stackebrandt E. (New York: Springer), 159–196.

[ref3] Broadhurst D. Goodacre R. Reinke S. N. Kuligowski J. Wilson I. D. Lewis M. R. . (2024). Guidelines and considerations for the use of system suitability and quality control samples in mass spectrometry assays applied in untargeted clinical metabolomic studies. Metabolomics 20:72. doi: 10.1007/s11306-024-02145-0 29805336 PMC5960010

[ref4] Chen Y. Lin Y. Zhu J. Zhou J. Lin H. Fu Y. . (2024). Transcriptomic analysis of nitrogen metabolism pathways in *Klebsiella aerogenes* under nitrogen-rich conditions. Front. Microbiol. 15:1323160. doi: 10.3389/fmicb.2024.1323160, 38500581 PMC10945327

[ref5] Chen H. Wang S. Guo R. Zhang L. Xu J. (2026). Multi-omics and stable isotopic reveal coordinated carbon-nitrogen metabolic reprogramming sustaining diatom cellular homeostasis under CO₂ limitation. Mar. Environ. Res. 215:107843. doi: 10.1016/j.marenvres.2026.107843, 41520592

[ref6] Chen Y. Zhong J. Li B. Dai W. Yang Z. Huang C. . (2023). Exploring the nitrogen removal capacity of *Klebsiella aerogenes* B23 isolated from shrimp farm wastewater: heterotrophic nitrification and aerobic denitrification. Aquac. Int. 32, 1453–1471. doi: 10.1007/s10499-023-01224-2

[ref7] Corbet C. Pinto A. Martherus R. Santiago de Jesus J. P. Polet F. Feron O. (2016). Acidosis drives the reprogramming of fatty acid metabolism in cancer cells through changes in mitochondrial and histone acetylation. Cell Metab. 24, 311–323. doi: 10.1016/j.cmet.2016.07.003, 27508876

[ref8] Dang H. Chen C.-T. A. (2017). Ecological energetic perspectives on responses of nitrogen-transforming chemolithoautotrophic microbiota to changes in the marine environment. Front. Microbiol. 8:1246. doi: 10.3389/fmicb.2017.01246, 28769878 PMC5509916

[ref9] Dawson H. M. Connors E. Erazo N. G. Sacks J. S. Mierzejewski V. Rundell S. M. . (2023). Microbial metabolomic responses to changes in temperature and salinity along the western Antarctic peninsula. ISME J. 17, 2035–2046. doi: 10.1038/s41396-023-01475-0, 37709939 PMC10579395

[ref10] Delmont T. O. Pierella Karlusich J. J. Veseli I. Fuessel J. Eren A. M. Foster R. A. . (2022). Heterotrophic bacterial diazotrophs are more abundant than their cyanobacterial counterparts in metagenomes covering most of the sunlit ocean. ISME J. 16, 927–936. doi: 10.1038/s41396-021-01135-1, 34697433 PMC8941151

[ref11] Díez J. López-Lozano A. Domínguez-Martín M. A. Gómez-Baena G. Muñoz-Marín M. C. Melero-Rubio Y. . (2023). Regulatory and metabolic adaptations in the nitrogen assimilation of marine picocyanobacteria. FEMS Microbiol. Rev. 47:fuac043. doi: 10.1093/femsre/fuac043, 36323406

[ref12] Fan L. Sun F. (2024). Nitrogen metabolism potential in biofilm microbial communities: potential applications in the mariculture wastewater treatment. Aquac. Eng. 104:102387. doi: 10.1016/j.aquaeng.2023.102387

[ref13] Feng Y. Feng J. Shu Q. L. (2018). Isolation and characterization of heterotrophic nitrifying and aerobic denitrifying *Klebsiella pneumoniae* and *Klebsiella variicola* strains from various environments. J. Appl. Microbiol. 124, 1195–1211. doi: 10.1111/jam.13703, 29356236

[ref14] Foyer C. H. Noctor G. Hodges M. (2011). Respiration and nitrogen assimilation: targeting mitochondria-associated metabolism as a means to enhance nitrogen use efficiency. J. Exp. Bot. 62, 1467–1482. doi: 10.1093/jxb/erq453, 21282329

[ref15] Gika H. G. Theodoridis G. A. Earll M. Wilson I. D. (2012). A QC approach to the determination of day-to-day reproducibility and robustness of LC–MS methods for global metabolite profiling in metabonomics/metabolomics. Bioanalysis 4, 2239–2247. doi: 10.4155/bio.12.212, 23046266

[ref1212] Gilcreas F. W. (1966). Standard methods for the examination of water and waste water. Am J Public Health Nations Health. 56, 387–388. doi: 10.2105/AJPH.56.3.387 5948695 PMC1256912

[ref16] Goulitquer S. Potin P. Tonon T. (2012). Mass spectrometry-based metabolomics to elucidate functions in marine organisms and ecosystems. Mar. Drugs 10, 849–880. doi: 10.3390/md10040849, 22690147 PMC3366679

[ref17] Guo L. Li L. Zhou S. Xiao P. Zhang L. (2024). Metabolomic insight into regulatory mechanism of heterotrophic bacteria nitrification-aerobic denitrification bacteria to high-strength ammonium wastewater treatment. Bioresour. Technol. 394:130278. doi: 10.1016/j.biortech.2023.130278, 38168563

[ref18] Hamed S. M. Tan U. Mohamed M. Y. A. Khalifa A. AbdElgawad H. (2025). Synergistic interaction of *Micromonospora* sp. and chitosan nanoparticles drive metabolic shifts for enhancement chickpea yield and seed quality. Int. J Biol Macromol 322:146879. doi: 10.1016/j.ijbiomac.2025.146879, 40816384

[ref19] He Y. T. Wang F. Yu S. Y. Xie C. X. Yuan L. Li Z. H. . (2026). Shift of nitrogen metabolism drives the evolution of multidrug resistance in wastewater microbiomes under long-term fluoride stress. Water Res. 299:125904. doi: 10.1016/j.watres.2026.125904, 41967247

[ref20] Huang K. Bai H. Meng C. Kashif M. Wei Z. Tang Z. . (2025). Deciphering the ammonia transformation mechanism of a novel marine multi-stress-tolerant yeast, *Pichia kudriavzevii* HJ2, as revealed by integrated omics analysis. Appl. Environ. Microbiol. 91, e0221124–e0221124. doi: 10.1128/aem.02211-24, 40338088 PMC12175507

[ref21] Huang F. Pan L. He N. Ban Q. (2022). The nitrite reductase encoded by nirBDs in *Pseudomonas putida* Y-9 influences ammonium transformation. Front. Microbiol. 13:982674. doi: 10.3389/fmicb.2022.982674, 36312953 PMC9597696

[ref22] Huang X. Weisener C. G. Ni J. He B. Xie D. Li Z. (2020). Nitrate assimilation, dissimilatory nitrate reduction to ammonium, and denitrification coexist in *Pseudomonas putida* Y-9 under aerobic conditions. Bioresour. Technol. 312:123597. doi: 10.1016/j.biortech.2020.123597, 32506044

[ref23] Inabe K. Miichi A. Matsuda M. Yoshida T. Kato Y. Hidese R. . (2021). Nitrogen availability affects the metabolic profile in cyanobacteria. Meta 11:867. doi: 10.3390/metabo11120867, 34940625 PMC8707274

[ref24] Kuypers M. M. M. Marchant H. K. Kartal B. (2018). The microbial nitrogen-cycling network. Nat. Rev. Microbiol. 16, 263–276. doi: 10.1038/nrmicro.2018.9, 29398704

[ref25] Li Y. Li C.-X. Lin W. Wang S.-S. Zhang W.-X. Jiang Y.-M. . (2021). Full evaluation of assimilatory and dissimilatory nitrate reduction in a new denitrifying bacterium *Leclercia adecarboxylata* strain AS3-1: characterization and functional gene analysis. Environ. Technol. Innov. 23:101731. doi: 10.1016/j.eti.2021.101731

[ref26] Liebeke M. Lalk M. (2014). *Staphylococcus aureus* metabolic response to changing environmental conditions – a metabolomics perspective. Int. J. Med. Microbiol. 304, 222–229. doi: 10.1016/j.ijmm.2013.11.017 24439195

[ref27] Miller-Fleming L. Olin-Sandoval V. Campbell K. Ralser M. (2015). Remaining mysteries of molecular biology: the role of polyamines in the cell. J. Mol. Biol. 427, 3389–3406. doi: 10.1016/j.jmb.2015.06.020, 26156863

[ref28] Moreno-Cabezuelo J. Á. Gómez-Baena G. Díez J. García-Fernández J. M. (2023). Integrated proteomic and metabolomic analyses show differential effects of glucose availability in marine *Synechococcus* and *Prochlorococcus*. Microbiol. Spectrum 11, e03275–e03222. doi: 10.1128/spectrum.03275-22, 36722960 PMC10100731

[ref29] Mulaudzi M. S. Nephali L. P. Tugizimana F. (2025). AI-integrated metabolomics maps functional divergence of microbial consortia in field-grown maize. Plant Cell Rep. 44:211. doi: 10.1007/s00299-025-03600-z, 40936028 PMC12426160

[ref30] Nagamani S. C. S. Erez A. Lee B. (2012). Argininosuccinate lyase deficiency. Genet. Med. 14, 501–507. doi: 10.1038/gim.2011.1, 22241104 PMC3709024

[ref31] Ren J. Tang J. Min H. Tang D. Jiang R. Liu Y. . (2024). Nitrogen removal characteristics of novel bacterium *Klebsiella* sp. TSH15 by assimilatory/dissimilatory nitrate reduction and ammonia assimilation. Bioresour. Technol. 394:130184. doi: 10.1016/j.biortech.2023.130184, 38086459

[ref32] Romero F. M. Maiale S. J. Rossi F. R. Marina M. Ruíz O. A. Gárriz A. (2018). “Polyamine metabolism responses to biotic and abiotic stress,” in Polyamines, Methods in Molecular Biology, eds. Alcázar R. Tiburcio A. F. (New York, NY: Springer), 37–49. 10.1007/978-1-4939-7398-9_3 29080153

[ref33] Sardans J. Gargallo-Garriga A. Urban O. Klem K. Walker T. W. N. Holub P. . (2020). Ecometabolomics for a better understanding of plant responses and acclimation to abiotic factors linked to global change. Meta 10:239. doi: 10.3390/metabo10060239, 32527044 PMC7345909

[ref34] Schiffer T. A. Lundberg J. O. Weitzberg E. Carlström M. (2020). Modulation of mitochondria and NADPH oxidase function by the nitrate-nitrite-NO pathway in metabolic disease with focus on type 2 diabetes. BBA Mol Basis Dis 1866:165811. doi: 10.1016/j.bbadis.2020.165811, 32339643

[ref35] Senior P. J. (1975). Regulation of nitrogen metabolism in *Escherichia coli* and *Klebsiella aerogenes*: studies with the continuous-culture technique. J. Bacteriol. 123, 407–418. doi: 10.1128/jb.123.2.407-418.1975, 238954 PMC235743

[ref36] Shaw D. R. Terada A. Saikaly P. E. (2024). Future directions in microbial nitrogen cycling in wastewater treatment. Curr. Opin. Biotechnol. 88:103163. doi: 10.1016/j.copbio.2024.103163, 38897092

[ref37] Smith S. R. Dupont C. L. McCarthy J. K. Broddrick J. T. Oborník M. Horák A. . (2019). Evolution and regulation of nitrogen flux through compartmentalized metabolic networks in a marine diatom. Nat. Commun. 10:4552. doi: 10.1038/s41467-019-12407-y, 31591397 PMC6779911

[ref38] Tomulescu C. Moscovici M. Lupescu I. Stoica R. M. Vamanu A. (2021). A review: *Klebsiella pneumoniae*, *Klebisellaoxytoca* and biotechnology. Rom. Biotechnol. Lett. 26, 2567–2586. doi: 10.25083/rbl/26.3/2567.2586

[ref39] Trautwein K. Hensler M. Wiegmann K. Skorubskaya E. Wöhlbrand L. Wünsch D. . (2018). The marine bacterium *Phaeobacter inhibens* secures external ammonium by rapid buildup of intracellular nitrogen stocks. FEMS Microbiol. Ecol. 94:fiy154. doi: 10.1093/femsec/fiy154, 30124819 PMC6122490

[ref40] Wu N. Yang M. Gaur U. Xu H. Yao Y. Li D. (2016). Alpha-ketoglutarate: physiological functions and applications. Biomol. Ther. 24, 1–8. doi: 10.4062/biomolther.2015.078, 26759695 PMC4703346

[ref41] Yan M. Firkins J. Guo J. Relling A. Yu Z. (2026). Genome-resolved multi-omics provide new insights into microbial nitrogen utilization by the rumen microbiota. Microbiome 14:152. doi: 10.1186/s40168-026-02422-9, 41987215 PMC13196213

[ref42] Yayo J. Rydzak T. Kuil T. Karlsson A. Harding D. J. Guss A. M. . (2023). The roles of nicotinamide adenine dinucleotide phosphate reoxidation and ammonium assimilation in the secretion of amino acids as byproducts of *Clostridium thermocellum*. Appl. Environ. Microbiol. 89:e0175322. doi: 10.1128/aem.01753-22, 36625594 PMC9888227

[ref43] Yoshidome D. Kuze K. Ichiyanagi A. Yoshida A. Kosono S. Nishiyama M. (2025). Increased L-glutamate production from gaseous nitrogen using *Klebsiella pasteurii* NG13 with modified citrate synthase. Appl. Microbiol. Biotechnol. 109:275. doi: 10.1007/s00253-025-13646-4, 41419653 PMC12718243

[ref44] Yuan J. Doucette C. D. Fowler W. U. Feng X. Piazza M. Rabitz H. A. . (2009). Metabolomics-driven quantitative analysis of ammonia assimilation in *E. coli*. Mol. Syst. Biol. 5:302. doi: 10.1038/msb.2009.60, 19690571 PMC2736657

[ref45] Zheng Q. Wang Y. Lu J. Lin W. Chen F. Jiao N. (2020). Metagenomic and metaproteomic insights into photoautotrophic and heterotrophic interactions in a *Synechococcus* culture. mBio 11, e03261–e03219. doi: 10.1128/mBio.03261-19, 32071270 PMC7029141

[ref46] Zhou Y. Li Q. Yang X. Wang L. Li X. Liu K. (2024). Mitigating high-temperature stress in peppers: the role of exogenous NO in antioxidant enzyme activities and nitrogen metabolism. Horticulturae 10:906. doi: 10.3390/horticulturae10090906, 30654563

