# Supplementary figures and images for: Adaptive reprogramming of carbon–nitrogen metabolism in Klebsiella aerogenes under nitrate-rich conditions
Source: Front Microbiol. 2026 Jun 17;17:1859352. doi: 10.3389/fmicb.2026.1859352 (PMC13319891; doi:10.3389/fmicb.2026.1859352)

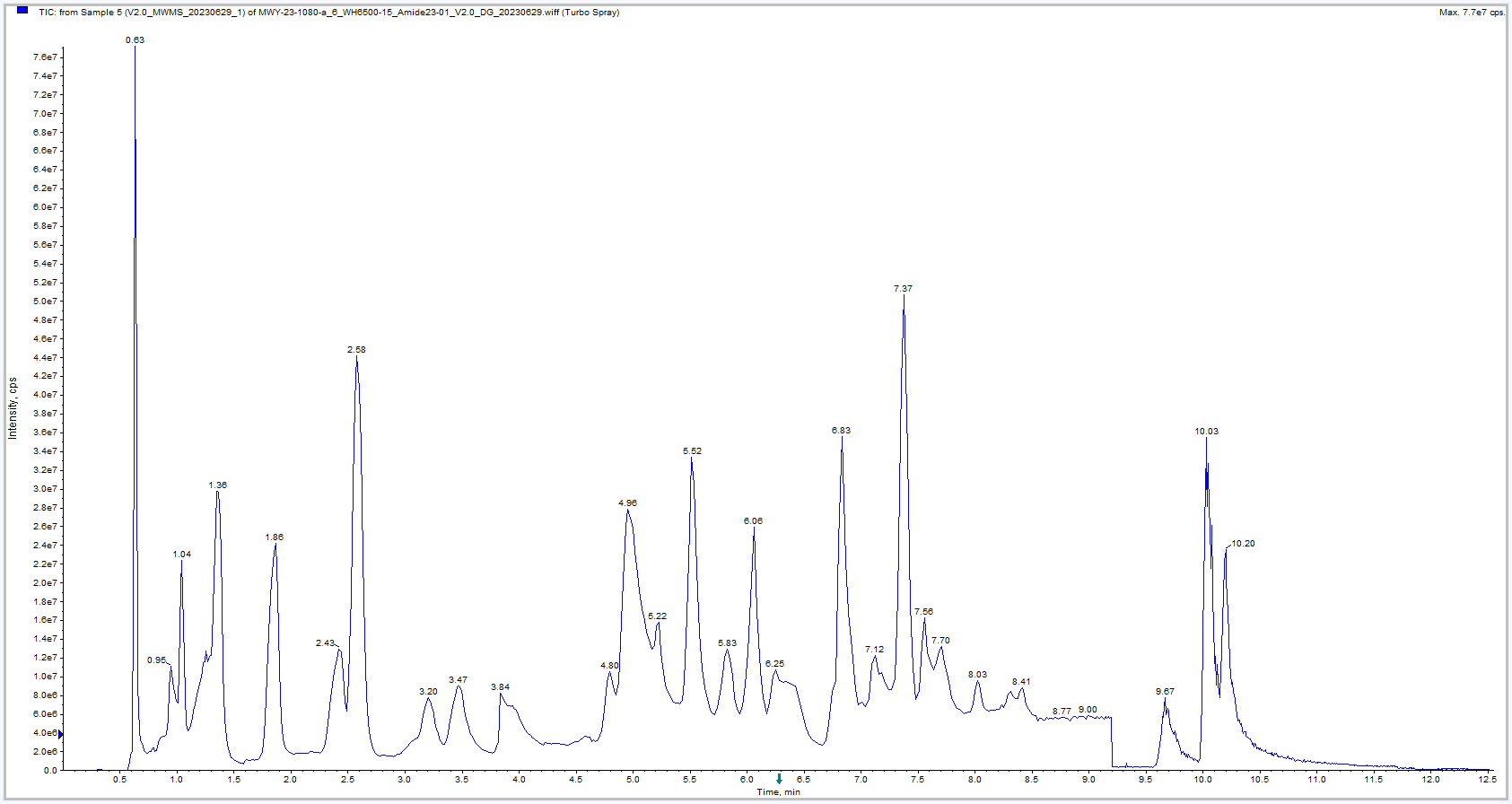

Supplement: Supplementary file 1 [file Image_1.png]

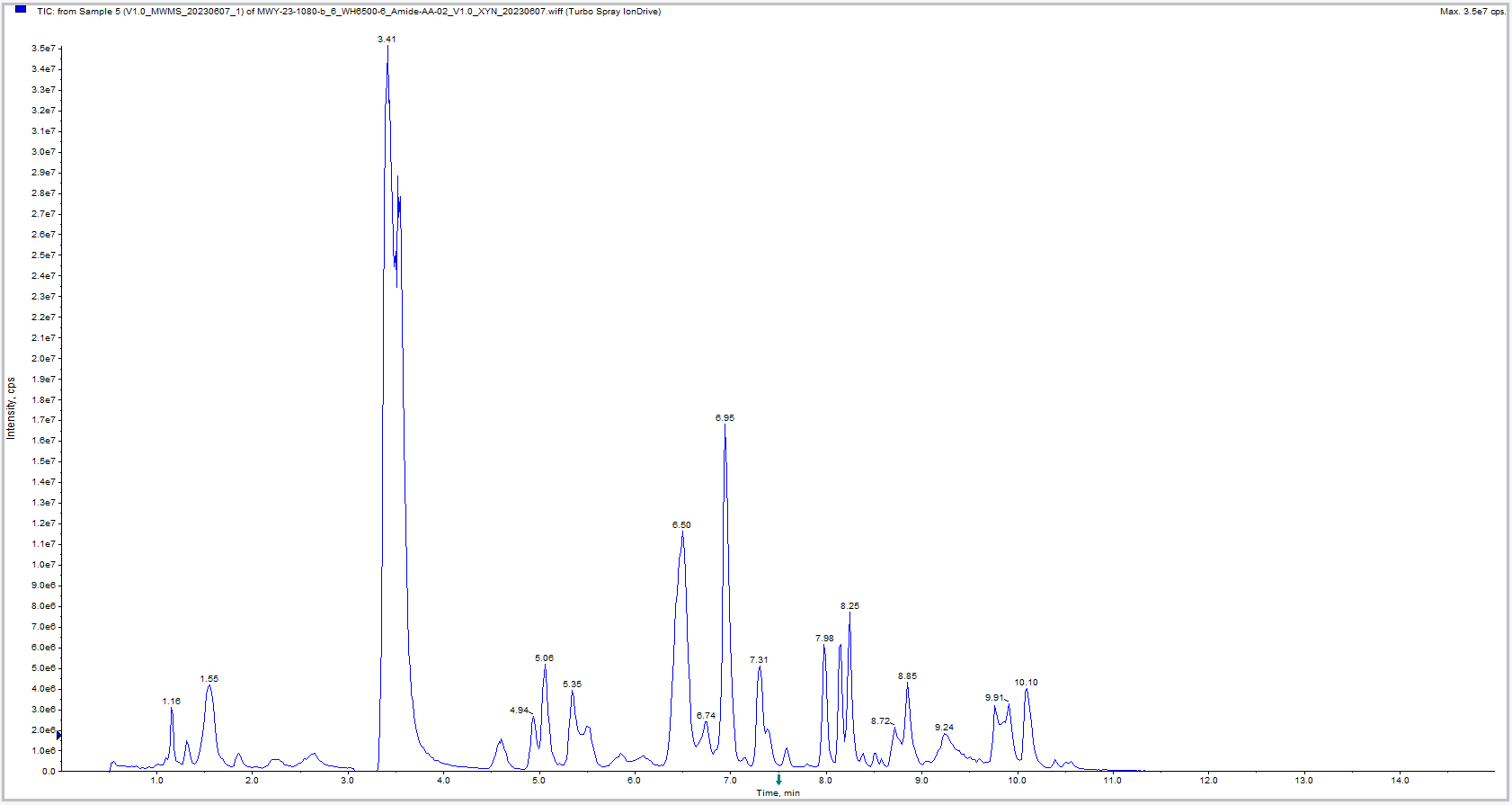

Supplement: Supplementary file 2 [file Image_2.png]

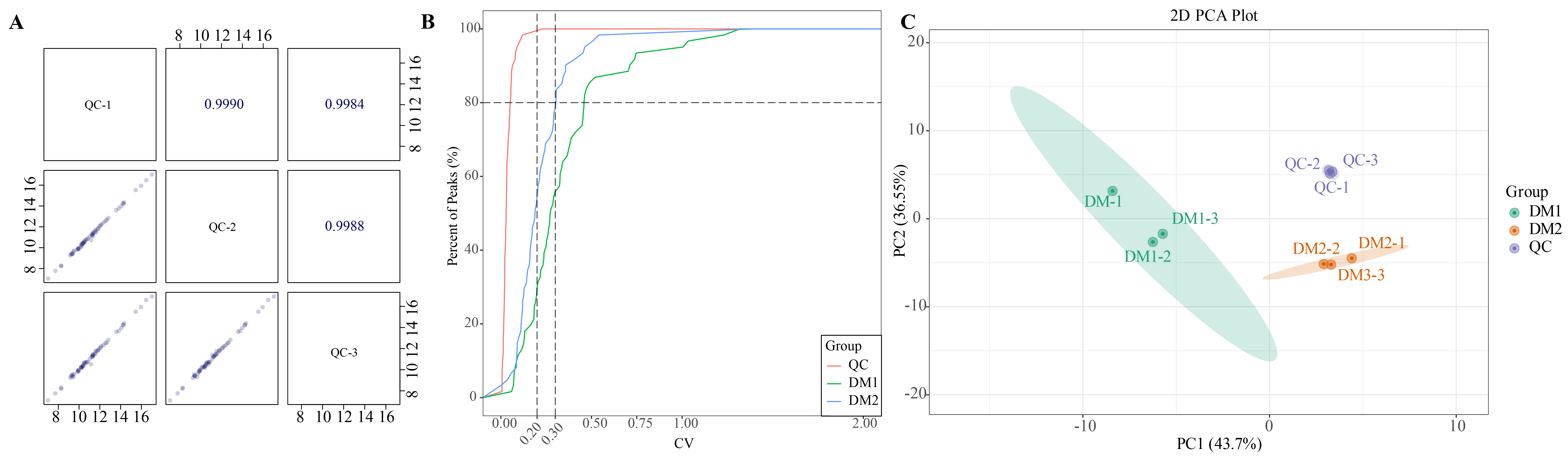

Supplement: Supplementary file 3 [file Image_3.png]

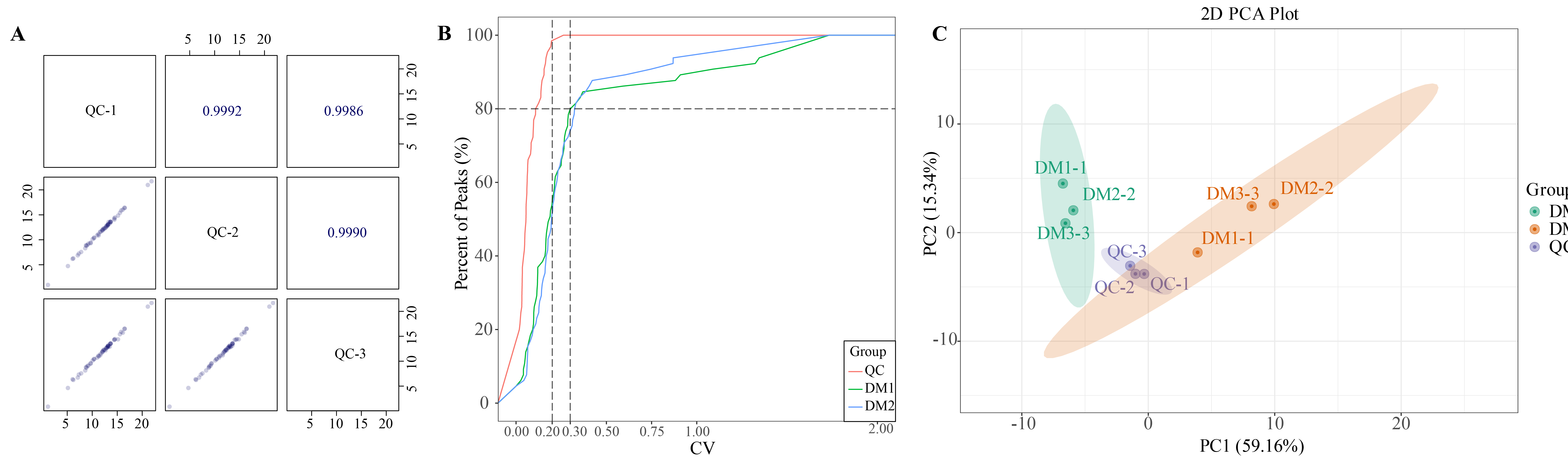

Supplement: Supplementary file 4 [file Image_4.png]

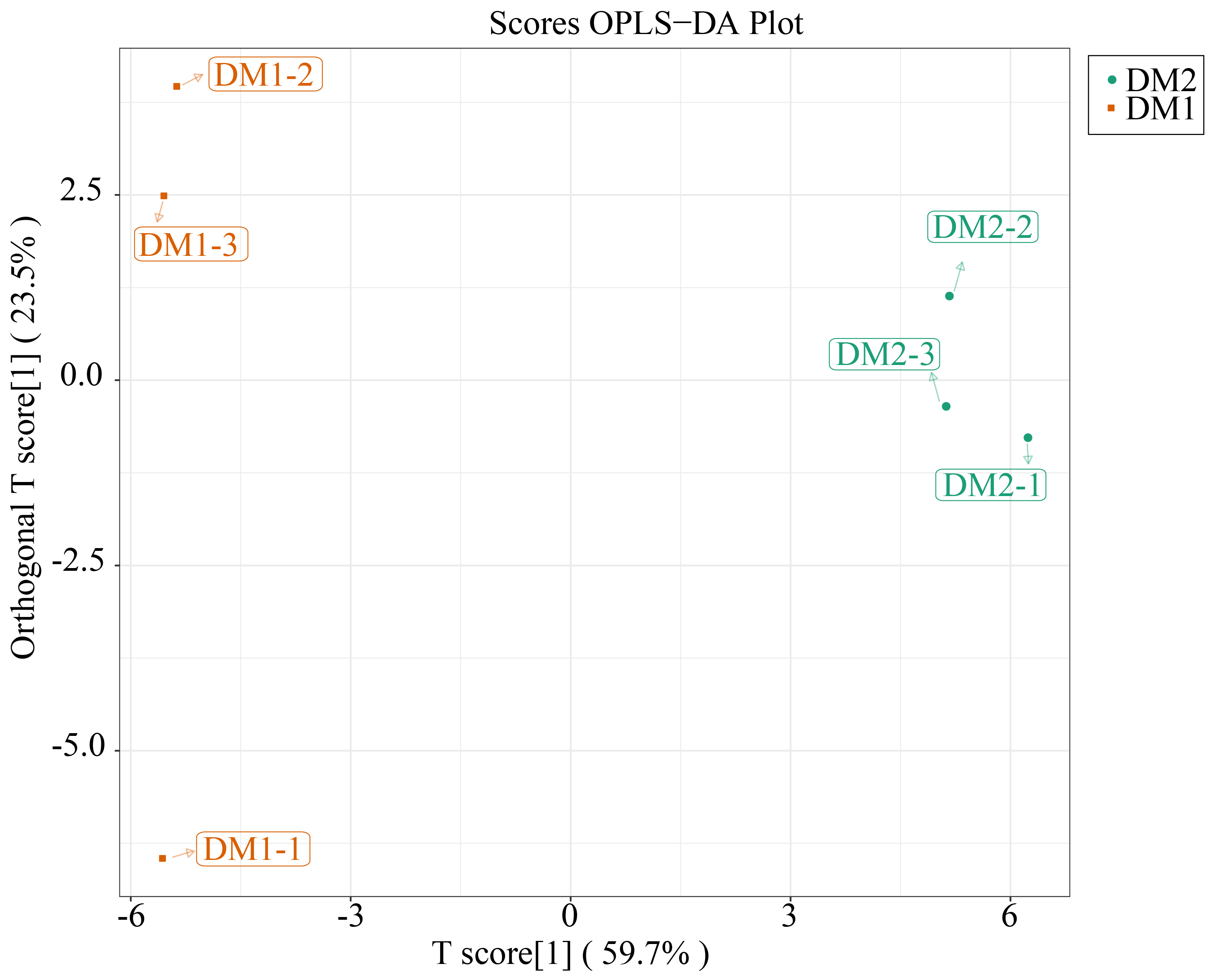

Supplement: Supplementary file 5 [file Image_5.png]
